# Nursing Students’ Relational Skills with Elders Improve through Humanitude Care Methodology

**DOI:** 10.3390/ijerph17228588

**Published:** 2020-11-19

**Authors:** Rosa Cândida Carvalho Pereira Melo, Paulo Joaquim Pina Queirós, Luiza Hiromi Tanaka, Liliana Vanessa Lúcio Henriques, Hugo Leiria Neves

**Affiliations:** 1 Health Sciences Research Unit: Nursing (UICISA: E), Nursing School of Coimbra (ESEnfC), 3046-851 Coimbra, Portugal; pauloqueiros@esenfc.pt (P.J.P.Q.); lilianahenriques@esenfc.pt (L.V.L.H.); hugoneves@esenfc.pt (H.L.N.); 2 Department of Collective Health, Paulista School of Nursing, Federal University of São Paulo, São Paulo 04024-002, Brazil; luiza.hiromi1@gmail.com

**Keywords:** nursing education, interpersonal relationships, humanization of assistance, patient-centered care, nurse–patient relations, old age assistance, Humanitude

## Abstract

Nursing students have difficulties interacting with cognitively impaired elders. This study aimed to identify students’ difficulties in interacting with elders, the causes of the difficulties in interacting with elders, the strategies used to reduce these difficulties, and the importance attributed to the Structured Sequence of Humanitude Care Procedures (SSHCP). It also aimed to assess the contribution of the Humanitude Care Methodology (HCM) to the development of interaction skills in nursing students. An exploratory descriptive study with a quasi-experimental design was conducted with a sample of 64 nursing students during their hospital clinical training. A control group (usual training) and an experimental group (HCM training) were used. Data were collected through a questionnaire applied at baseline and follow-up. Content analysis, chi-square tests, and Student’s t-tests were performed. The main difficulty identified was caring for agitated and confused elders. Difficulties were associated with a lack of theoretical–practical teaching, the clinical training context, lack of experience, and personality traits. HCM impacted positively on the development of students’ interaction skills. This study shows that HCM is an innovative methodology in nursing education that will allow for moving from an instrumental and technicist education into a more humanized training capable of transforming care.

## 1. Introduction

One of the main focus areas in nursing is the interaction with the patient, as it is essential for achieving excellence in care delivery [1]. Thus, as nurses’ attitudes and behaviors have a direct impact on the quality of care, nursing students must develop relational skills from the moment they begin their studies, which will help them understand what it means to help others [1,2,3] and, consequently, contribute to the humanization of care.

The humanization of care was proposed by several theorists, such as Carl Rogers, who was a humanist psychologist and the founder of client-centered therapy [4]; Joyce Travelbee, who developed the human-to-human relationship model [5]; and Hildegard Peplau, who developed the interpersonal relations theory [6]. Although several nursing theories focus on the individual and on interpersonal relations, nursing education still focuses on instrumental techniques and procedures rather than on relational practice [1]. In addition, there is a lack of intentionality and integration of these topics in clinical settings, leading to ritualistic practices in education [4] and difficulties in interacting with elders with behavioral changes [7,8].

Growing numbers of elders and age-associated comorbidities, especially dementia, have become a challenge for nursing students because they have to learn how to communicate with these patients effectively [7,8,9,10,11]. Therefore, the development of relational skills during clinical training in institutions that care for elders is essential for preparing this new generation of professionals. It is important to change the attitudes of these nursing students towards caring for cognitively impaired elders to ensure that they will have improved practices as future nurses. This development will boost a differentiated care strategy capable of responding to complex chronic health needs associated with aging [9,10,11,12].

Studies show that undergraduate programs should include elder care training in a more methodological and systematized way [9,10,11,12,13,14]. Therefore, nursing students should develop a care relationship with elders, especially those with dementia, and they should also be provided with methodologies to operationalize and systematize the relationship according to scientific and ethical principles. To do so will transform care delivery into an enjoyable moment for both patients and caregivers [12,13]. Despite the need for a methodology that systematizes and combines both clinical care and a humanized approach to elder care, no studies have been published on this topic, particularly in the area of nursing education.

To systematize relational care, the Humanitude Care Methodology (HCM), which was developed by Yves Gineste and Rosette Marescotti [13,14,15] in 1979, follows a Structured Sequence of Humanitude Care Procedures (SSHCP). The purpose of the SSHCP is to create a positive relationship and achieve a sense of wellbeing for the patient and the caregiver during care delivery [13,14,15,16,17,18].

The SSHCP consists of five dynamic and consecutive stages: the first stage, the “pre-preliminaries”, aims at preparing the environment and the patient for the initial approach while respecting the patient’s privacy, freedom, and autonomy; the second stage, the “preliminaries” correspond to the beginning of a positive relationship with the patient and to the first contact with the patient through gaze, speech, and touch; the third stage is the “sensory circle” or provision of care, which is the coherent organization of sensory inputs (sight, sound, touch, and smell), leading to a sensation of wellbeing; the fourth stage, called “emotional consolidation”, allows for the creation of a positive impression on emotional memory, providing the patient with positive feedback about their self-care efforts and progress, and thanking them for the time spent together; and the last stage, “appointment”, which involves saying goodbye and scheduling the next appointment, while avoiding feelings of abandonment and neglect [13,15,17,18].

This methodology is based on the relational pillars (gaze, speech, and touch) that should be taught to nursing students so they can use them effectively with intention in care delivery [13,14,15,16,17,18]. Thus, nursing students need to develop skills relating to the way they look at the patient, taking into account the following aspects: horizontal, looking at the same level, demonstrating equality, so that the person does not feel inferior; axial, looking straight at the person, demonstrating honesty; long, to build a relationship of trust; and progressively close, so that the person feels that, at that moment, the caregiver is concerned about meeting their needs [13,14,15,16,17,18]. Regarding speech, it is essential to train the voice to achieve the following characteristics: melodic, calm, rhythmic, and deep tone, using positive and pleasant words to talk to clients. Touch should have the following characteristics: gradual, starting in socially accepted areas (hand, arms, and shoulders); wide, covering a larger contact area; continuous and slow, avoiding sudden movements [13,14,15,16,17,18].

Although the humanization of care is not a new or novel topic, its implementation is complex and often guided by empiricism and trivialization [19]. Evidence has shown the impact of the operationalization and systematization of this concept when caring for elders in several countries, such as France, Switzerland, Belgium, Germany, Portugal, Japan, and the United States [15,16,20]. However, research on nursing education is still scarce, and there is a need to assess nursing students’ difficulties in caring for elders with behavioral changes.

Therefore, this study aims to: identify nursing students’ difficulties in interacting with elders; identify the causes of those difficulties; identify the strategies used for overcoming those difficulties; identify the importance attributed to the SSHCP; and assess the impact of the implementation of the HCM on nursing students’ interaction skills. This study aims to contribute to the transformation of pedagogical practices, offering students relational techniques to develop professional attitudes that can facilitate nursing practices.

## 2. Materials and Methods

A quasi-experimental study was conducted using the non-equivalent groups pre-test–post-test design. The population was composed of 360 second-year undergraduate students from a Portuguese nursing school, receiving their clinical training at the medicine and neurology wards of a central hospital. These wards have, on average, 30 beds, and the majority of the patients are aged 80 years or older with some type of dementia-manifested behavioral changes, such as agitation, confusion, and care refusal.

The sample consisted of 64 students who were assigned to an experimental group (EG) that received HCM training (EG = 32) and a control group (CG) that received conventional training (CG = 32). Inclusion criteria for the EG were: being second-year nursing students; having attended the optional course unit “Caring with Humanitude”; receiving their clinical training at a central hospital for ten weeks in medicine or neurology wards; and applying the HCM during their clinical training. For the CG, the inclusion criteria were being second-year nursing students; having no knowledge of HCM; receiving their clinical training at a central hospital for ten weeks in medicine or neurology wards; and performing the relational procedures usually taught at the nursing school.

The CG sample was randomly selected from the students who met the inclusion criteria using the randomization function (RAND) of Microsoft Excel. The EG consisted of the 32 students who attended the optional course unit “Caring with Humanitude”, and no student dropped out. This course unit was taught at the nursing school before the clinical training, in a total of 27 h of theoretical classes, by a teacher who previously took HCM training. This teacher also supervised the clinical training. At the theoretical classes, the philosophical concepts and principles of Humanitude and the HCM were addressed by reflecting upon and discussing videos using the HCM. Technical–relational procedures were trained based on the SSHCP, and field trips were organized to institutions that care for elders. In the clinical training, the following on-the-job training strategies were used: relational skills training; weekly reflection on the difficulties in interacting with elders with dementia who were agitated and who refused care; and the elaboration of reflective essays in the 5th and 10th weeks of clinical training by the students to reflect on the contribution of the HCM towards reduce their difficulties in interacting with elders with behavioral changes.

Data were collected in both groups at two different moments: at baseline (that is, at the beginning of clinical training, which is the 3rd week after integration in the wards and contact with the patients), and follow-up (that is, at the end of clinical training at the 10th week). At baseline, 64 students participated (EG = 32; CG = 32), no student dropped out, and all students answered all questions. At follow-up, 60 students participated (EG = 31; CG = 29), one EG student did not answer the questions, and three CG students did not answer the questions.

Students were asked to fill in a questionnaire with closed-ended and open-ended questions. Closed-ended questions were aimed at the sociodemographic characterization of the sample and the identification of difficulties in interacting with elders. Open-ended questions included: “What are the causes of the difficulties identified?” and “What strategies did you use to reduce the difficulties in interacting with elders?” At the end of the clinical training, the EG students answered the following question: “Did the HCM, through its SSHCP, facilitate the development of interaction skills?” This questionnaire followed the theoretical framework of Humanitude and previous studies [8,21] that aimed to identify students’ difficulties and the causes of such difficulties, as well as the strategies used to overcome/minimize them. A questionnaire validated by Simões, Salgueiro and Rodrigues [13] was used to determine the importance attributed to the SSHCP. The questionnaire consists of 31 items that represent a Humanitude care procedure. It assesses the importance attributed by the student to each procedure on a 4-point Likert scale, where 1 corresponds to “Not important at all” and 4 to “Extremely important”. Data were collected in writing, at a time previously agreed with the students, at the beginning (3rd week) and end (10th week) of clinical training in a classroom. Both sessions lasted approximately 20 min. Data were collected by the team of researchers, who were professors at the nursing school.

Data from the closed-ended questions were processed using IBM SPSS software, version 27 (IBM Corp, Armonk, New York, United States of America). The chi-square test was applied for the analysis of the association between variables. Group differences for the SSHCP scores were assessed through the use of Student’s t-test and represented through the test statistics (t), *p*-value (*p*), effect size through Cohen’s *d* (*d*), and the Cohen’s *d* 95% Confidence Interval. All inferential test requirements were assessed prior to the inferential test selection. A 0.05 probability of type I errors (α) was set for all inferential analyses.

The categorical content analysis was used for open-ended questions based on the principles of homogeneity, completeness, exclusivity, objectivity, and pertinence for the identification of categories, according to Bardin’s theoretical framework [22]. Content analysis began with an analysis and exploration of the collected data, resulting in the identification of the themes that emerged from the questions. Data related to each theme were organized into categories and subcategories. Specific codes were assigned to recording units with a specific semantic content previously established by the researcher. These recording units were organized based on the coding of the answer segments, whose content constituted a core unit and was used for the frequency count.

Theoretical data saturation in each category was assumed when no new themes emerged from the data [23]. Five steps were used to identify theoretical saturation [23]: Step 1—recording of raw data from the answers to the open-ended questions and written transcription of the answers; Step 2—immersion in data, with a fluctuating reading of the data; Step 3—compilation of individual analyses and distribution of the categories using colorimetric coding; Step 4—arrangement of the categories, subcategories, and statements in a table to find regularity in the findings according to the categories and consistency in the statements; Step 5—confirmation of theoretical data saturation by identifying the lack of new elements in each category.

The validity and reliability of content analysis were ensured by two coders who were experts in qualitative research and who validated the findings. Participants were also allowed to provide their feedback on the findings. Qualitative and quantitative data triangulation was performed through the analysis of the difficulties and their causes, as well as of the strategies used by the students at the beginning and end of their clinical training, to reduce these difficulties and to assess the impact of the HCM on the EG.

Regarding research ethics, the following steps were completed. The President of the Nursing School authorized the study, and it was approved by the ethics committee of the Health Sciences Research Unit: Nursing (UICISA: E) of the Nursing School of Coimbra, Portugal, under favorable opinion no. 302-09/2015. Each student signed an informed consent form and was briefed about the voluntary nature of their participation and that they could withdraw from the study without any penalty or consequences. The researchers, who were professors at the nursing school and who accompanied the students during the clinical training period, had access to anonymized material, which ensured confidentiality. This study respects the Declaration of Taipei [24] and the Declaration of Helsinki [25]. All rights regarding data protection were respected, permission has been obtained, and there are no copyright issues.

## 3. Results

### 3.1. Sample Characterization

The sample consisted mostly of female students (EG 87.5%; CG 75.8%). The mean age was 20.09 years (±2.26) in the EG and 20.36 years (±1.99) in the CG, ranging from 19 to 31 years. Both groups were similar in gender and age.

### 3.2. Students’ Difficulties in Interacting with Elders

The main difficulties identified by all students in interacting with elders at baseline and follow-up were: communicating with non-communicative elders (baseline 68.8%; follow-up 50.0%), caring for agitated and confused elders (baseline 54.7%; follow-up 48.3%), communicating with elders with aphasia (baseline 45.3%; follow-up 55.0%), initiating communication with elders (baseline 37.5%; follow-up 21.7%), and caring for elders who refuse care (baseline 23.4%; follow-up 31.7%).

#### Comparison of the Difficulties Identified by EG and CG Students

Table 1 shows that CG students identified more difficulties in interacting with elders than EG students, both at baseline and follow-up.

- At baseline, the EG identified fewer difficulties than the CG in “caring for agitated and confused elders” (χ^2^ = 7.630; *p* = 0.006) and “communicating with non-communicative elders” (χ^2^ = 4.655; *p* = 0.031). No inter-variable correlations were found in the remaining difficulties identified (*p* > 0.05)
- At follow-up, the EG identified fewer difficulties than the CG in “caring for agitated and confused elders” (χ^2^ = 6.637; *p* = 0.010), “communicating with elders with aphasia” (χ^2^= 6.877; *p* = 0.009), and “initiating communication with elders” (χ^2^= 8.748; *p* = 0.003).

### 3.3. Causes of the Difficulties Identified by Students during Their Clinical Training

From the content analysis of the question “What are the causes of the difficulties identified in clinical training?”, in the CG and EG, four categories emerged: inexperience, deficit in theoretical–practical teaching, clinical training context, and personal traits.

Table 2 shows the categories, subcategories and recording units of the difficulties experienced by EG and CG students at baseline and follow-up. Although the EG students identified fewer difficulties than those in the CG, more recording units (96) regarding the causes of the difficulties were identified in the EG than in the CG (74).

#### 3.3.1. Causes of Difficulties Associated with Inexperience

Inexperience was one of the most common causes of difficulties in interacting with elders in both groups of students. In the category “inexperience”, the subcategories “lack of experience” and “first contact with reality” emerged.

With regard to the lack of experience, the students reported: “That lack of experience was a major difficulty that I felt…” (S69). During clinical training, students had their first contact with reality, which was perceived as a difficulty: “…when I first had contact with the reality of care, I was very scared and afraid of not being able to communicate…” (S71), “…we should contact with the reality of care earlier on…” (S18).

#### 3.3.2. Causes of Difficulties Associated with the Deficit in Theoretical–Practical Teaching

In the category “deficit in theoretical–practical teaching”, the following subcategories emerged: deficit of knowledge about communication, difference between theory and practice, deficit of relational techniques training, and procedure-focused simulation.

According to the students, in this category, the most common cause of difficulties was the deficit of relational techniques associated with the deficit of knowledge about communication: “…communication is taught in a very theoretical way, they teach very abstract concepts, lack of training on how it is done in practice…” (S19), “…relational techniques training that help us to communicate with elders who are agitated, confused, or refusing care…” (S4).

Another cause for the difficulties was the difference between what they had learned in theory and the actual practice: “…there are differences between what is taught and valued in theory and what we see in clinical practice, (...) care is focused a lot on the tasks that must be performed and not on the relationship with the clients...” (S3).

The students also emphasized that simulations in theoretical teaching were focused on technical and instrumental procedures, which was also identified as a cause of their difficulties,: “…in laboratory classes, the technical procedures are simulated using manikins, and communication is not trained, there’s a difference between using simulators or operating on a real person…” (S53), “…on manikins, training is very focused on the technical and instrumental aspects and does not value the communication and relationship component…” (S19).

The deficit in theoretical–practical teaching was the most identified category in the EG as a cause of difficulties (baseline = 23; follow-up = 19).

#### 3.3.3. Causes of Difficulties Associated with the Clinical Training Context

In the category “clinical training context”, the following subcategories emerged: deficit of Humanitude in the professionals, lack of respect from the professionals, and understaffing.

In the EG, the most frequent cause of the difficulties associated with the clinical training context was the deficit of professionals’ training in Humanitude: “…it would be important for nurses to have Humanitude training so that they can learn to respect the characteristics of each person…” (S35), “…it is clear that they are not always sensitive to the Humanitude care methodology, they focus more on tasks…” (S451).

There were also reports of a lack of respect from the professionals towards the students while implementing what they learned about Humanitude: “…sometimes there is a lack of understanding and respect by the nurses, they are always in a hurry, we have to keep up with their pace…” (S5). The lack of respect from the professionals towards the patients was also reported: “the lack of respect for the clients’ time, performing their care for them, without promoting their decision, which makes them increasingly dependent…” (S50).

In the CG, understaffing, especially the nurse–patient ratios, was the most common cause of the difficulties in this category: “…lack of nurses, they are few and are always in a hurry…” (S78).

#### 3.3.4. Causes of Difficulties Associated with Personality Traits

In the category “personal characteristics”, the following subcategories emerged: shyness, lack of self-confidence, anxiety, and fear.

Lack of self-confidence was the most valued subcategory associated with personality traits: “I feel a lack of self-confidence in interacting with elders because I don’t know how to start and end a conversation…” (S79); “…in the case of elders who are agitated and refuse care, I don’t know what to do, I have low confidence…” (S97).

Another cause of the difficulties in interacting with elders was anxiety: “I feel very anxious about failing and clients rejecting me…” (S76). Shyness also caused interaction difficulties: “I’m a little shy…” (S9); “…embarrassed at the beginning…” (S12).

Fear was another cause of difficulties in interacting with elders: “I’m afraid because I don’t know how to perform the techniques correctly and I’m afraid to fail in my clinical training” (S77); “…fear of bothering clients with my presence…” (S76), “…fear of hurting the person or not being able to perform the technical procedures…” (S78).

Personality traits was the most identified category in the CG as a cause of difficulties (baseline = 19; follow-up = 11).

### 3.4. Strategies Used by Students to Reduce Difficulties in Interacting with Elders

The content analysis of the answers to the question “What strategies did you use to reduce the difficulties in interacting with elders?” obtained 107 recording units and three categories emerged: application of the HCM, training/education, and development of personal skills.

Table 3 shows the categories, subcategories, and recording units of strategies used by EG and CG students to reduce difficulties at baseline and follow-up. The EG identified more strategies (80) to overcome the difficulties, both at the beginning and end of the clinical training, compared to the CG (27).

#### 3.4.1. Strategies Used by EG Students to Reduce Difficulties in Interacting with Elders

In the EG, regarding the strategies used to overcome the difficulties at the beginning and end of clinical training, three categories emerged: application of the HCM, training/education, and development of personal skills.

- In the category “application of the HCM”, the following subcategories emerged: knowledge of the elders, Humanitude pillars, and technical–relational procedures. Knowledge of the elders, their life history, and the words that calm them down prevent agitation, according to this statement: ”…knowing the elders’ past, as well as the words that calm them down or the negative words that agitate them and that should be avoided” (S14). The proper use of the Humanitude pillars such as gaze, speech, and touch, as well as availability and active listening, were also emphasized: “…adequately using gaze, speech, and touch, making them company, taking the time to listen to them…” (S5); “…in elders who cannot communicate verbally, the strategy used was self-feedback, using both a predictive speech and a descriptive speech that allowed us to overcome the embarrassing silence” (S27).
- In the category “training/education”, the following subcategories emerged: training in “Caring with Humanitude”, self-training, relational techniques training, and practical context. Attendance at the optional course unit “Caring with Humanitude” was emphasized by these students: “…the optional course unit ’Caring with Humanitude’ taught me how to initiate communication, how to look patients in the eye, how to speak and touch them” (S18); “…it taught me how to speak calmly and encourage the patient to communicate, how, when and where to touch the elders…” (S1). The need for self-training, relational techniques training, and knowledge of the HCM was also mentioned: “We need to continue the HCM training on our own and to train more the relational techniques that we’ve learned” (S13); “Improve and train verbal and non-verbal communication” (S20). In the practical context, the need for nurses to have HCM training and not to follow bad examples was also evidenced: “Teaching HCM techniques to nurses so that we can apply what we have learned, so that there are ‘good examples’ in care” (S2), “…and we can avoid learning from the ‘bad examples’ that we observe in daily practice…” (S19).
- In the category “development of personal skills”, the following subcategories emerged: self-confidence, emotional management, and persistence. In this category, the importance of improving communication was emphasized as a means of increasing students’ self-confidence, emotional management, and acceptance of the patient: “…I must learn how to communicate so that the client accepts me and so that I can feel more confident manage my anxiety and the fear of interacting better” (S12); “Have a smile on my face and keep calm” (S15); “Should be persistent and not give up on the client” (S51).

#### 3.4.2. Strategies Used by CG Students to Reduce Difficulties in Interacting with Elders

In the CG, regarding the strategies used to reduce difficulties at the beginning and end of clinical training, two categories emerged: training/education and development of personal skills.

- In the category “training/education”, the following subcategories emerged: self-training, relational techniques training, and practical context. The need for self-training as a strategy to reduce difficulties was mentioned: “Reading articles about therapeutic communication” (S78). Students also emphasized the importance of relational techniques training: “Acquiring experience and skills that allow me to be better…” (S79). Regarding practical context, students identified that following a practical role model was valuable “Using nurses’ strategies…” (S72).
- In the category “development of personal skills”, the following subcategories emerged: self-confidence, emotional management, and persistence. Students identified the importance of self-confidence as a strategy to reduce difficulties at the beginning and end of clinical training: “Have more confidence in what I do, remain calm, and not give up” (S79). At baseline, emotional management was also mentioned: “…communicate more with the clients to overcome difficulties…” (S77). One student identified persistence as a strategy used to reduce difficulties at follow up: “...try to develop strategies because I feel like I need to be more relaxed to perform a more complex technical procedure, (...) keep calm and concentrated during the procedures…” (S76).

### 3.5. Importance Attributed to the SSHCP Dimensions

The results suggest that EG students attribute greater importance to the SSHCP dimensions than CG students. Table 4 shows that a statistically significant difference was found at the beginning of clinical training between the CG and the EG regarding the importance attributed to the SSHCP dimensions, particularly the pre-preliminaries (t = 3.384; *p* = 0.001; *d* = 0.853), the preliminaries (t = 2.655; *p* = 0.010; *d* = 0.669), and the appointment (t = 3.034; *p* = 0.004; *d* = 0.759), with the EG attributing greater importance to these dimensions than the CG. At the end of clinical training, a statistically significant difference was found between both groups regarding the importance attributed to the preliminaries (t = 2.594; *p* = 0.012; *d* = 0.682), and the appointment (t = 2.460; *p* = 0.018; *d* = 0.647), with the EG attributing greater importance to these dimensions than the CG.

### 3.6. Impact of the HCM on the Development of Interaction Skills

All EG students (100%) answered yes to the question posed at the end of clinical training: “Did the HCM, through its SSHCP, facilitate the development of interaction skills?” CG students did not answer this question because they had no contact with this care methodology.

## 4. Discussion

In this study, nursing students had their first contact with the reality of care in the internal medicine and neurology wards of a central hospital, which have a high prevalence of elders with neurocognitive disorders. Students experienced interaction difficulties, anxiety, and fear because they did not feel well-prepared [7,8,21].

The main difficulties in both groups of students at the beginning and end of clinical training were: interacting with elders who cannot communicate verbally and caring for elders who are agitated, confused, and refuse care. The results are in line with those found in other studies involving nursing students, showing that they are not well-prepared to care for elders with cognitive impairment and experience difficulties when challenging their technicist care practice [1,7,8,21].

The difficulties in communicating with and caring for elders were more evident in CG students. Statistics showed that this group had more difficulties in caring for agitated and confused elders at the beginning and end of clinical training (*p* < 0.05). This difference in both groups may result from the intervention performed with EG students, as these students attended the optional course unit “Caring with Humanitude” where they received HCM training that operationalizes and systematizes the relationship with the patient, facilitating communication [13,14,15,16,17,21,26].

EG students perceived fewer difficulties in providing care to elders with behavioral changes, namely agitation and refusal of care. The integration of relational techniques recommended by the HCM, such as the intentionality attributed to gaze (axial, horizontal, long, and close), speech (melodious and soft tone of voice), the use of positive words, and the negotiation of care, among others, contributed to the reduction of agitation behaviors and refusal of care [13,14,15,16,17,18,26].

Overall, although EG students had fewer difficulties in interacting with elders during their clinical training than CG students, they were able to identify more causes of their difficulties, possibly because they were more aware of and more capable of reflecting on their own practices, showing empathy towards the patient. This reflects the need for a greater investment in relational techniques teaching and training [1,8,21,27,28,29].

At the beginning and end of the clinical training, the students’ difficulties focused on the deficit of theoretical–practical teaching, namely due to the deficit of relational techniques training. Simulation-based learning is mainly guided by the technicist model, focusing on instrumental procedures rather than on the relational dimension [8,21,30]. This focus reflects a training gap in the current nursing education [28] contrary to the current guidelines that promote a greater emphasis on the interaction with the patient [30,31]. For the humanization of assistance, simulation must also integrate relational skills in addition to the training of psychomotor skills [8]. Critical reflection and questioning about the practice should also be used [21].

The clinical training context was also identified as a complicating factor, which may be attributed to the professionals’ lack of Humanitude training. This lack of training has led to the development of a task-centered care practice, which does not respect the time the client and the student require. Therefore, students who experience disrespectful environments during their training are unlikely to develop humanized and reflective attitudes after finishing their studies [19]. A humanized care environment must be promoted during the students’ training [19,30] so they can learn from good role models and not from ‘bad examples’. Thus, a technicist care practice that disregards relational skills will increase the difficulties in interacting with patients [8,30,31,32].

Inexperience, especially because it was the students’ first contact with the reality of clinical practice, was another difficulty that should be considered in nursing education because it causes anxiety in students [8,28]. The relationship should be intentional and professional, using various strategies such as the demonstration and training of relational techniques [1,30,33] developed through realistic simulation that integrates the relational component [31].

The main difficulties for CG students were personal traits, namely lack of confidence, fear, and anxiety. These results are in line with those found in other studies [1,7,8,28], showing that students’ fear of verbal and non-verbal communication impacts patient perception, leading to resistance behaviors, agitation, and refusal of care [31,32]. Thus, it becomes essential to implement programs to promote students’ personal development skills [1,7,8], intervening intentionally in their training process to promote the vital relational skills for the humanization of care [1,2,8,30,31,32] and to reduce these communication difficulties.

EG students demonstrated an awareness and integration of solid strategies to reduce the difficulties in interacting with elders. The application of the HCM, namely giving a sense of purpose to the initial approach to the patient so as to avoid agitation [13,17,18,21,26], and using relational pillars such as an adequate way of establishing eye contact, communicating verbally, and touching the patient, were part of these strategies. These Humanitude care procedures are systematized and arranged in a logical order to facilitate communication and interaction with elders, contributing to the operationalization of the humanization of care [13,18,21,26,32] for these students.

Other strategies were demonstrating availability, giving the patient time to perform self-care, and active listening, because the humanization of care relies on speaking and listening to others [30,32].

The use of self-feedback was identified as a strategy for interacting with elders with aphasia, namely predictive speech (that is, saying what we will do to prevent surprise approaches and agitation) [17,18]. As for descriptive speech, which means communicating what we are doing to promote multisensory stimulation, it helped overcome the awkward silence [21]. During care delivery to non-communicative patients, it is of the utmost importance to use self-feedback for the person to feel integrated into the care process, avoiding defensive behaviors, and facilitating care [17,21].

Although CG students identified more difficulties in interacting with elders than those in the EG, they identified fewer strategies. They focused on more generic aspects, namely gaining experience and using experienced nurses’ strategies. This type of learning based on routinized practice models, without reflection on and in action, can lead to a mechanized and de humanized practice [30,31]. On the other hand, these students emphasized the technical procedures, disregarding the relational component, and the need to distance themselves from the client and focus on the task. Given this situation, nursing education should integrate a sense of purpose to the operationalization and systematization of the relational component in clinical practice, promoting a meaningful learning experience for students [1,21].

EG students identified more solid strategies to minimize their difficulties than CG students, possibly due to the Humanitude training. The meetings throughout clinical training to reflect on action and in action and the demonstration and training of relational techniques allowed for raising more awareness and reinforcing the ability to identify strategies for reducing their difficulties. The individual awareness of students and the use of integrated teaching models are essential to move from a technicist education to a critical–reflexive education [30,31]. This will contribute to the humanization of health professionals’ training [30,33], especially nurses’ training, as the process of humanization requires professionals to have scientific and ethical knowledge and be prepared to deliver humanized care [30,34].

Thus, as communication in clinical settings has its specificities, specific communication training should be integrated into nursing education programs or programs in other health areas where professionals interact more closely with people with cognitive impairment [34]. Communication training should focus on the conceptual and theoretical aspects and be developed in an integrated and systematized way, including a strong practical component in clinical settings [21,29,34].

EG students attribute more importance to the SSHCP dimensions than those in the CG, which means that the theoretical training in Humanitude care promoted a greater valorization of Humanitude care procedures in care delivery. These findings are in line with those found in other studies [13,26] regarding the importance of training in this area for a greater awareness and intentionality in Humanitude care procedures. In this study, a statistically significant difference was found at the beginning of clinical training between the CG and the EG, with higher scores in the EG, namely in the pre-preliminaries and preliminaries relating to the preparation of the appointment and the initial approach to the patient. These differences were also observed in the procedures related to the end of the relationship and the scheduling of the next appointment, relating to the avoidance of feelings of abandonment and neglect, and the increased acceptance of the next moment of care delivery [13,26].

This study has some limitations, such as the small sample size, increasing the risk of non-representativeness and preventing data generalization. Another limitation was the difficulty in controlling all variables so that the CG had the same characteristics and conditions as the EG. As different clinical training contexts influence nursing students’ experiences, this factor may influence the results of this study. Nevertheless, to minimize this limitation, special attention was put on the selection of the clinical training contexts to ensure similar characteristics, namely being in the same hospital and in similar wards (neurology and internal medicine). Further studies should be conducted in other clinical training contexts and with more students.

The small number of studies conducted and published on this topic in the area of education made it difficult to compare the results. This study showed the need for research in this area, evidencing promising results towards a more efficient implementation of this methodology in nursing education and clinical practice, especially given the increasing number of elders who require adequate, individualized care. To address this research gap, we recommend the development of a multicentric randomized controlled trial involving nurses and other health professionals who provide care to patients with cognitive impairment.

## 5. Conclusions

Elder care delivery requires the development of scientific and ethical practice. For nursing practice to meet the requirements of complex humanized care to this population, innovative teaching methodologies to enhance relational skills must be implemented.

This study evidenced how nursing students can benefit from the development of relational skills through training, incorporating a sense of purpose on the use of communication strategies. Achieving this will require a change in perspective in nursing education. To change from a historical emphasis on technical procedures to more humanized and relationship-centered teaching requires evidence and awareness of all the actors of this new reality. Therefore, the implementation of the HCM can be an innovative educational tool in nursing education, contributing to a humanized training capable of transforming clinical practice, influencing the wellbeing of the caregiver and the patient. The integration of this methodology in nursing education will help future nurses acquire the abovementioned sense of purpose, leading to a world where elders are respected and cared for as human beings.

There is no doubt that HCM training will change nursing care delivery. Above all, it will promote the delivery of humanized care, with a clear impact on the quality of life of this highly vulnerable population.

## Figures and Tables

**Table 1:** Chi-square test results for the difficulties identified by students in the experimental group (EG) and control group (CG) at baseline and follow-up.

Students’ Difficulties	Moment of Assessment		EG		CG		Total		χ^2^	*p*
			*n*	%	*n*	%	*n*	%		
Initiating communication with elders	Baseline	Yes	10	31.3	14	43.8	24	37.5	1.067	0.302
		No	22	68.8	18	56.3	40	62.5		
	Follow-up	Yes	2	6.5	11	37.9	13	21.7	8.748	0.003
		No	29	93.5	18	62.1	47	78.3		
Communicating with non-communicative elders	Baseline	Yes	18	56.3	26	81.3	44	68.8	4.655	0.031
		No	14	43.8	6	18.8	20	31.3		
	Follow-up	Yes	12	38.7	18	62.1	30	50.0	3.270	0.071
		No	19	61.3	11	37.9	30	50.0		
Communicating with elders with aphasia	Baseline	Yes	11	34.4	18	56.3	29	45.3	3.090	0.079
		No	21	65.6	14	43.8	35	54.7		
	Follow-up	Yes	12	38.7	21	72.4	33	55.0	6.877	0.009
		No	19	61.3	8	27.6	27	45.0		
Communicating with agitated and confused elders	Baseline	Yes	12	37.5	23	71.9	35	54.7	7.630	0.006
		No	20	62.5	9	28.1	29	45.3		
	Follow-up	Yes	10	32.3	19	65.5	29	48.3	6.637	0.010
		No	21	67.7	10	34.5	31	51.7		
Caring for elders who refuse care	Baseline	Yes	7	21.9	8	25.0	15	23.4	0.087	0.768
		No	25	78.1	24	75.0	49	76.6		
	Follow-up	Yes	7	22.6	12	41.4	19	31.7	2.447	0.118
		No	24	77.4	17	58.6	41	68,3		

χ^2^, Chi-square test.

**Table 2:** Causes of the difficulties experienced by EG and CG students at baseline and follow-up.

Categories	Subcategories	Moment of Assessment	Recording Units	
			EG	CG
Inexperience	Lack of experience	Baseline	12	7
		Follow-up	11	9
	First contact with reality	Baseline	7	6
		Follow-up	1	1
Deficit in theoretical–practical teaching	Deficit of knowledge about communication	Baseline	3	4
		Follow-up	1	0
	Difference between theory and practice	Baseline	6	3
		Follow-up	3	3
	Deficit of relational techniques training	Baseline	11	1
		Follow-up	12	0
	Procedure focused simulation	Baseline	3	2
		Follow-up	3	1
Clinical training context	Deficit of Humanitude in the professionals	Baseline	5	0
		Follow-up	4	0
	Lack of respect from the professionals	Baseline	2	0
		Follow-up	1	1
	Understaffing	Baseline	1	3
		Follow-up	1	3
Personal traits	Shyness	Baseline	3	1
		Follow-up	0	3
	Lack of self-confidence	Baseline	1	7
		Follow-up	1	5
	Anxiety	Baseline	1	2
		Follow-up	1	1
	Fear	Baseline	2	9
		Follow-up	0	2
Total			96	74

**Table 3:** Strategies used by EG and CG students to reduce difficulties at baseline and follow-up.

Categories	Subcategories	Moment of Assessment	Recording Units	
			EG	CG
Application of the HCM	Knowledge of the elders	Baseline	3	0
		Follow-up	1	0
	Humanitude Pillars	Baseline	12	0
		Follow-up	4	0
	Technical–relational procedures	Baseline	6	0
		Follow-up	13	0
Training/education	Training in “Caring with Humanitude”	Baseline	4	0
		Follow-up	6	0
	Self-training	Baseline	3	5
		Follow-up	4	3
	Relational techniques training	Baseline	8	5
		Follow-up	5	5
	Practical context	Baseline	2	1
		Follow-up	0	0
Development of personal skills	Self-confidence	Baseline	2	4
		Follow-up	3	1
	Emotional management	Baseline	1	2
		Follow-up	1	0
	Persistence	Baseline	0	0
		Follow-up	2	1
Total			80	27

HCM, Humanitude care methodology.

**Table 4:** Results of the Student’s t-test for independent samples regarding the importance attributed to the Structured Sequence of Humanitude Care Procedures (SSHCP) dimensions by CG and EG students at baseline and follow-up.

SSHCP Dimensions	Moment of Assessment	Groups	*n*	Mean	SD	t	*p*	ES	ES	
									95% CI	
Pre-preliminaries	Baseline	EG	32	3.59	0.42	3.384 ^a^	0.001	0.853 ^b^	0.333	1.366
		CG	31	3.20	0.49					
	Follow-up	EG	30	3.68	0.38	1.483 ^a^	0.144	0.390 ^b^	−0.132	0.908
		CG	28	3.53	0.42					
Preliminaries	Baseline	EG	32	3.65	0.31	2.655 ^a^	0.010	0.669 ^b^	0.159	1.174
		CG	31	3.45	0.28					
	Follow-up	EG	30	3.76	0.21	2.594 ^a^	0.012	0.682 ^b^	0.149	1.209
		CG	28	3.60	0.27					
Sensory circle	Baseline	EG	30	3.64	0.36	1.028 ^a^	0.308	0.268 ^b^	−0.246	0.779
		CG	29	3.54	0.38					
	Follow-up	EG	29	3.72	0.28	1.798 ^a^	0.078	0.472 ^b^	−0.052	0.992
		CG	29	3.57	0.35					
Emotional consolidation	Baseline	EG	32	3.69	0.46	0.661 ^a^	0.511	0.167 ^b^	−0.329	0.661
		CG	31	3.61	0.44					
	Follow-up	EG	30	3.77	0.34	1.822 ^a^	0.074	0.474 ^b^	−0.045	0.990
		CG	29	3.60	0.37					
Appointment	Baseline	EG	32	3.70	0.42	3.034 ^a^	0.004	0.759 ^b^	0.248	1.263
		CG	32	3.35	0.49					
	Follow-up	EG	30	3.80	0.30	2.460 ^a^	0.018	0.647 ^b^	0.121	1.169
		CG	29	3.49	0.60					

SD, standard deviation; ES, effect size; CI, confidence interval; *n*, number of participants; ^a^ independent t-test, significant at α = 0.05 (two-tailed); ^b^ Cohen’s d.
